# Developing a framework to describe stigma related to cervical cancer and HPV in western Kenya

**DOI:** 10.1186/s12905-022-01619-y

**Published:** 2022-02-11

**Authors:** Ramya Ginjupalli, Rachel Mundaden, Yujung Choi, Emily Herfel, Sandra Yvonne Oketch, Melissa H. Watt, Breandan Makhulo, Elizabeth Anne Bukusi, Megan Huchko

**Affiliations:** 1 Center for Global Reproductive Health, Duke Global Health Institute, 310 Trent Dr, Durham, NC 27710 USA; 2 Center for Microbiology Research, Kenya Medical Research Institute, Off Mbagathi Road, P.O. Box 54840 00200, Nairobi, Kenya; 3 Department of Population Health Sciences, University of Utah, Williams Building, Room 1N490, 295 Chipeta Way, Salt Lake City, UT 84108 USA; 4 Department of Obstetrics and Gynecology, Duke University, 2301 Erwin Road, Durham, NC 27710 USA

**Keywords:** HPV, Cervical cancer, Stigma, Kenya

## Abstract

**Background:**

Despite a high prevalence of human papillomavirus (HPV) and cervical cancer in low and middle-income countries, stigma remains an issue. Addressing HPV and cervical cancer stigma could significantly improve health outcomes for these conditions. The objective of this study was to identify the manifestations of stigma and their potential impacts on health-seeking behavior.

**Methods:**

Twenty-six in-depth interviews were conducted with women living with HIV, HIV-negative women, community health volunteers, and health care providers in Kisumu, Kenya in 2019. The interviews were designed to draw out existing attitudes or experiences related to stigma within the community. We conducted a thematic analysis of the interviews to identify internalized, anticipated, and discriminatory attitudes.

**Results:**

Within internalized attitudes, a prominent observed theme was a fear of death associated with a positive HPV test. This stemmed from a lack of understanding of differences between HPV and cervical cancer and posed a significant barrier for women deciding to seek screening or to continue with treatment. Discriminatory attitudes of community members, including assumptions of promiscuity, infidelity, or HIV status, were perceived to prevent women from accessing screening and treatment opportunities. The interviews also exhibited a limited awareness of HPV in this region, which may have contributed to a lack of enacted stigma towards people living with HPV or cervical cancer.

**Conclusion:**

Stigma has the potential to lead to decreased screening and treatment uptake through its drivers. This includes a decreased perception of personal risk due to a lack of knowledge, which results in increased HPV-risk behaviors. Future research must focus on creating and integrating stigma-reducing interventions, primarily to encourage women to seek out primary and secondary preventative measures.

**Supplementary Information:**

The online version contains supplementary material available at 10.1186/s12905-022-01619-y.

## Background

Cervical cancer, caused by human papillomavirus (HPV), disproportionately affects women in low and middle-income countries (LMICs), where 85% of global cervical cancer deaths occur [1]. The greatest burden of cervical cancer falls in East Africa [2]. High-risk HPV types contribute to 80% of cervical cancer cases and can be prevented through the use of HPV vaccination and cervical cancer screening [3]. Screening and vaccination for high-risk HPV are effective prevention strategies for cervical cancer. Vaccination can prevent up to 80% of cervical cancer cases, and is starting to become more widely available to adolescent girls in LMICs [4].

Even as vaccination access increases, screening will remain an essential tool to prevent cervical cancer. The World Health Organization (WHO) recommends HPV-based screening, as the most effective strategy to reduce cervical cancer incidence. Common examples of screening methods include Papanicolaou smear (pap smear), pap smear with HPV co-testing, HPV self-collection, and visual inspection with acetic acid and Lugol's iodine (VIA/VILI) [5]. With the availability of HPV vaccines and cost-effective screening technologies, the WHO in August 2020 called to action a strategic plan for eliminating cervical cancer [6]. While many high-income countries (HICs) are on target to achieve elimination in the next fifty years, vast disparities in screening, treatment, and access to vaccinations may make this ambitious goal impossible to achieve in LMICs.

While ensuring access, affordability, and quality of cervical cancer prevention services is paramount, the decision to utilize these services is influenced by knowledge, personal risk perception, and acceptability. All of these factors may be impacted by stigma related to HPV or cervical cancer. Stigma, which is defined as “labeling, stereotyping, separation, status loss, and discrimination” based on a specific identity or health condition can interfere with health-seeking behavior, resulting in delayed diagnosis and delay or avoidance of treatment [7, 8]. Individuals manifest negative messages or stereotypes about their condition and apply it to themselves in the form of internalized stigma [9]. Anticipated stigma is the belief that an individual will face future discrimination or prejudice from others [10]. HPV and cervical cancer stigma may have effects similar to HIV-related stigma, which has hampered the effectiveness of the HIV-response, including uptake of HIV testing [11, 12]. Studies have demonstrated that individuals with high levels of HIV-related stigma may resort to denial and avoidance over testing and disclosure, which affects disease progression or increases the risk of HIV transmission [13, 14].

In HICs and LMICs alike, personal beliefs, stigma, and misconceptions about reproductive health and sexuality may lead to a lack of screening and HPV vaccine uptake. HPV may be stigmatizing due to the method of transmission and site of infection, as seen with previous research regarding HIV stigma [15, 16]. In countries such as India, there is stigma related to cancer within female reproductive organs, including the breast and cervix [17]. In South Africa, studies have shown that “labeling” based on physical manifestations of cancer symptoms has led to anticipated discrimination and delayed treatment for individuals with cancer [18]. Literature suggests that HPV stigma exists within the United States, which has led to low HPV vaccine uptake, even when resources are available [19]. There is a gap in understanding the character and effects of HPV stigma and health-related outcomes in LMICs, as most of the research has been conducted in HICs.

Our prior work in this region demonstrated an association between loss-to-follow-up, inadequate knowledge, and stigma [20]. Given the increasing availability of both HPV testing and vaccination, it is important to understand the health beliefs and experiences that may serve as barriers to care. We sought to explore attitudes that may reflect internalized or anticipated stigma and to develop a framework of HPV and cervical cancer-related stigma among women in a high HIV prevalent area of western Kenya.

## Methods

A qualitative study was conducted to explore knowledge, attitudes, behaviors, and experiences related to HPV and cervical cancer. Study participants were women living with HIV, HIV-negative women, health care providers, and community health volunteers (CHVs). We used the data from in-depth interviews with these key stakeholders to develop a framework of HPV and cervical cancer-related stigma.

For recruitment, posters throughout the Family AIDS Care and Education Services (FACES) and Government of Kenya outpatient facilities in Kisumu, Kenya advertised “a study to explore what women think about cervical cancer and human papillomavirus.” The interviews were conducted in a FACES facility to ensure that women were not further stigmatized by participating in this study. FACES staff assisted with the identification of eligible women while the study team engaged their interest. Due to previous limited availability in resources within healthcare facilities, few participants would have had personal experience either getting screened or offering to screen. Fifteen women between the ages of 25-65 living with HIV and enrolled in care at FACES-supported sites were recruited to participate in the study. Five self-identified HIV-negative women between the ages of 25-65 and who were eligible for cervical cancer screening also participated in the study. For a provider’s perspective, three CHVs and three health care providers were interviewed as well. All in-depth interviews were conducted at the FACES clinics in Kisumu, Kenya between June and July 2019.

The interview guides were designed to elicit discriminatory attitudes, potential internalized stigma, and anticipated stigma. Discriminatory attitudes are the beliefs held by the community that perpetuates stigma such as promiscuity or unfaithfulness. Potential internalized stigma is the beliefs held by an individual that leads to self-prejudice, self-blame, embarrassment, and hopelessness. Lastly, anticipated stigma is the belief that an individual will face stigma, such as discrimination, harassment, and prejudice, from others.

Each woman was interviewed by a member of the research team in her preferred language (English, Luo, or Swahili) with interviews lasting approximately one hour. The questions asked to HIV-negative women and women living with HIV fell into the following categories: knowledge and experiences with HPV and cervical cancer, experiences and stigma surrounding HIV as compared to HPV, decision-making processes in health care, and challenges associated with living with HIV, HPV, or cervical cancer. Health care providers were asked a different set of questions surrounding HIV care, working with women living with HIV, HIV and HPV risks and treatments, cervical cancer and screening services, and challenges associated with an HIV, HPV, or cervical cancer diagnosis. Similar to health care providers, CHVs were interviewed on broad categories such as the role of CHVs and CHV training, working with women living with HIV, experiences and comfort with HPV and cervical cancer care, and challenges associated with an HIV, HPV, or cervical cancer diagnosis. A full list of the interview questions for each participant group can be found in Additional file 1: Appendix A.

The interviews were transcribed and then translated into English as necessary by the research team. A codebook was developed to identify themes such as stigmatizing attitudes, misinformation about HPV and cervical cancer, internalized attitudes, attitudes towards the screening process, etc. The full list of codes and definitions can be found in Additional file 2: Appendix B. The English transcripts were then coded using NVivo 12 software.

The team created a framework connecting *a priori* assumptions, existing literature on HIV, cervical cancer, and HPV stigma, and results from the thematic analysis to show how intrinsic factors related to HPV and cervical cancer played into culturally specific drivers of stigma. The team then used the existing and newly collected data to develop potential mitigation strategies for HPV and cervical cancer-related stigma.

This study was approved by the Ethical Review Boards at the Duke University School of Medicine (IRB# Pro00101931) and The Kenya Medical Research Institute Scientific and Ethical Review Unit (SERU #3894).

## Results

In June and July 2019, 26 women participated in in-depth interviews at the FACES clinics in Kisumu, Kenya. The participants consisted of 15 women living with HIV, 5 HIV-negative women, 3 community health volunteers, and 3 health care providers.

**Fig. 1:**
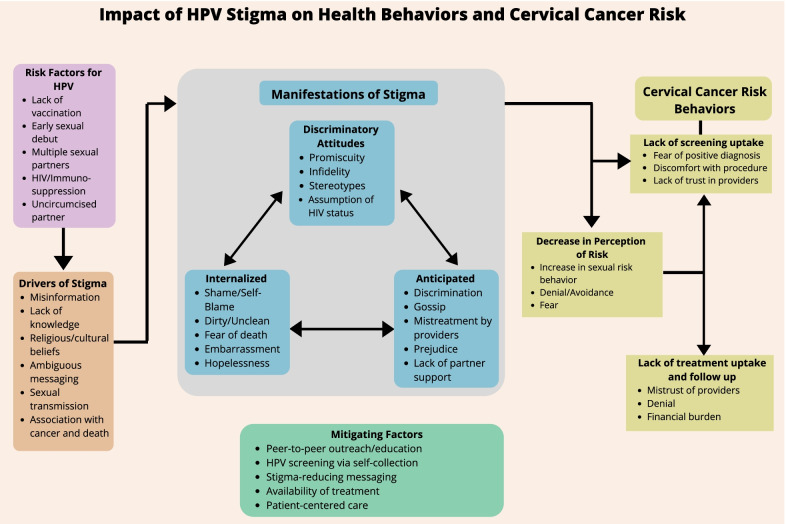
Perceived drivers, manifestations, and outcomes of stigma for HPV positivity or progression to cervical cancer from women living with HIV, HIV-negative women, community health volunteers, and health care providers in Kisumu, Kenya.

Discriminatory attitudes, potential internalized stigma, and anticipated stigma related to HPV and cervical cancer within the transcripts solidified the structure of our framework (Fig. 1). When not properly addressed, these manifestations of stigma may lower a woman’s cervical cancer risk perceptions and negatively impact her decision to utilize cervical cancer screening and/or treatment services. However, several mitigating factors may intervene to influence a woman's attitude, perception, and behavior toward pursuing cervical cancer screening and treatment. These mitigating factors may occur at any point of a woman’s cervical cancer continuum. The existing knowledge that guided the framework included risk factors for HPV, such as a lack of access to vaccinations, early sexual debut, multiple sexual partners, the increased risk among women living with HIV, and having sex with uncircumcised men.

Women were aware of some risk factors for HPV and cervical cancer, with the responses demonstrating that these risk factors were associated with potential drivers of stigma, including misinformation, lack of knowledge, religious/cultural beliefs, and ambiguous educational messaging by providers at the clinic.

### Risk factors for HPV

When asked “what causes someone to contract HPV,” most women believed that early sexual debut and having multiple sexual partners were the primary causes.

There is that HPV stigma when they disclose to another person. Some people have that knowledge, others don’t have that knowledge...people hear that multiple sexual partners or sex at an early age is what brings HPV. (Health care provider)

While these may be risk factors, they are not direct causes of HPV or cervical cancer. This misinterpretation further stigmatizes women with these risk factors.

Many women reported that if they heard someone had HPV and/or cervical cancer, they would immediately speculate that person has HIV as well.

If someone I know is diagnosed with HPV... the first question I will ask is...are you HIV positive? People tend to think that because of low immunity, we are prone to other diseases. (HIV negative woman)

### Drivers of stigma

One of the main drivers of HPV stigma was misinformation. Most of the interviewed subjects had a complete or almost complete absence of understanding about HPV and cervical cancer. Misinformation or lack of education may lead women to an improper understanding of the causes for HPV.

Cancer is caused by having multiple births … or by cigarettes. (Woman living with HIV)

Although some women had previously attended women’s health-related seminars to educate themselves about HPV, it was clear that for some women the ambiguous delivery of health information led to confusion and incorrect understanding about the mode of HPV transmission.

[If] I have [HPV], and I am seated. If someone else comes and sits here, she gets [HPV as well]. That is what I grasped [from education], I don’t know if I got it right (Woman living with HIV)

Similar to misinformation and lack of knowledge, ambiguous messaging or terminology during the health talks may have led to misinterpretation of the information. Without clear information at a level that these women could understand, the women were left to fill in the blanks, leading to further stigmatization and stereotyping.

They say the [germs] we get from men cause [HPV]. When [these germs] get in [the vagina], it starts growing... and eating up the area. [HPV] is the first sign and then the next one is [cervical] cancer (HIV negative woman)

Religious and cultural beliefs may also drive stigma, preventing women from seeking HPV and cervical cancer screening.

We have these religions that [believe] ….a speculum examination is a sin. According to their religion ... they should only be subjected to speculum examination when they are giving birth. (Community health volunteer)

Some women reported that religious leaders encouraged their followers to trust traditional medicine or the healing power of God rather than in modern medicine. If the women believe the screening services to be immoral and against their ideologies, they are not likely to take part.

They are told you should not take medicine because Jesus is the healer or because this medicine will make you infertile ... they are so inclined in their religion that they cannot see the benefit of getting a certain type of treatment...some people in the communities have myths and misconceptions about HPV and cervical cancer that it may be some witchcraft. They start seeking other avenues to clear the condition...even if they are brought to the hospital they decline treatment because of their ideologies.” (Community health volunteer)

The fact that HPV is a sexually transmitted disease also places a stigma on the disease itself and those who have it.

The fact that HPV is related to reproductive health, people will tend to think or people will just know or have in their mind that its about sex...so there is stigma. (HIV negative woman)

### Manifestations of stigma

#### Anticipated stigma from community members

There were multiple stigmatizing attitudes expressed by the community about an HPV-positive individual including promiscuity or unfaithfulness, since having multiple sexual partners is a risk factor for HPV.

Their partners should stick to faithfulness, not sleeping with multiple ladies, because those ladies may also be sleeping with other men. For me it is the same way for sleeping with multiple men. (HIV negative woman)

HPV-positive individuals are also assumed to have HIV, given the increased risk of HPV and cervical cancer among women living with HIV. Although this is a valid association, the assumption of an HIV diagnosis may lead to stigma, given the known stigmatization of HIV among many women.

If I hear someone is suffering from [cervical] cancer, number one I will think of death, number two I will think of what stage, and number three I will think of your HIV status. I would just be curious like a normal human being (HIV negative woman)

There was an absence of experienced or witnessed discrimination from cervical cancer or HPV. This contrasted vastly with the instances of experienced HIV stigma and discrimination we observed in the interviews, including unemployment due to HIV status, abandonment by partner, and domestic violence. Discrimination is typically linked to visible symptoms of an illness. Since there are minimal visible symptoms for HPV and cervical cancer, examples of discrimination based on symptoms alone were not found.

#### Potential internalized stigma

Women felt that if they tested positive for HPV and/or are diagnosed with cervical cancer, they would feel shame and self-blame associated with their diagnosis, especially if they were unfaithful in their relationship.

I should be blamed because I am the one who went looking for [sex] (Woman living with HIV)

Dirty, unhygienic, and unclean were words often associated with contracting HPV or cervical cancer in multiple interviews. These words carry negative connotations and can contribute to self-stigmatization for women living with HPV.

[To prevent contracting HPV] we can use condoms to prevent the [germs] from entering inside us. If we have sex with our men, we should maintain cleanliness, so to not be dirty. (Woman living with HIV)

Regarding a hypothetical diagnosis of HPV, most women immediately demonstrated a fear of death. This stemmed from the belief that HPV is the same disease as cervical cancer, reflecting a lack of knowledge or effective education, as HPV is treatable and not a death sentence.

There are difficulties they face … [some believe] that if diagnosed with cancer then all that awaits is death itself. (Woman living with HIV)

These women who believed that HPV is the same disease as cervical cancer also remarked how they would have feelings of hopelessness and despair if diagnosed with HPV.

I will just cry and know I am ready for death. I know I have cancer and I am dying soon because there is nothing that can be done. (HIV negative woman)

### Cervical cancer risk behaviors

#### Decrease in perception of risk

Some of the manifestations of stigma led to an overall decrease in the perception of personal risk, leading to risk-taking behaviors. For instance, long-term partners may not use barrier method contraceptives, due to their assumption that their partner is faithful.

You find that they didn’t test with their partners when they started dating. They don’t use contraceptives like condoms. [...] for women who are married or they have [...] the polygamous setup. You find that [these women in committed relationships] end up being infected unknowingly (Community health volunteer)

Infidelity could leave those in the relationship vulnerable to contracting HPV or other STDs. In the context of western Kenya, where polygamy is a practice accepted by some cultures, polygamy may also play a role in increasing HPV risk.

Due to ambiguous educational messaging related to HPV and cervical cancer prevention, some women believe that they are at a lower risk or no risk of contracting HPV if their partner is circumcised, leading to a potential for increased unprotected intercourse. While circumcision may make it harder for men to carry or transmit HPV to their partner, engaging in unprotected sex is still a risky behavior. Protection, regardless of circumcision status, should be encouraged during educational interventions.

[The way to prevent cervical cancer is] only if you have sex with someone who is circumcised or you use condoms (Woman living with HIV)

#### Lack of screening uptake

Avoidance of screening was another risk behavior that could be stigma-based. The observed fear of death related to an HPV diagnosis may lead some women to avoid testing. For women living with HIV, many were fearful of receiving an additional diagnosis of HPV. This leads to women refusing to be screened or tested for HPV in fear of receiving a positive diagnosis which may exacerbate their health burdens.

A woman would not want to get screened because she will be told that she has cancer and she will die. (Woman living with HIV)

#### Lack of treatment uptake and follow-up

Women also described stigma within a healthcare setting, suggesting a potential mistrust of providers and a decrease in health-seeking behaviors related to cervical cancer. Health care workers can also further drive stigmatization through misinformation, stereotypes, and making women feel “dirty” or “unclean.” If women believe they will be stigmatized within a facility for their HPV diagnosis, they will be less likely to seek treatment.

Those providing cancer services have attitude, but those attending to HIV patients are very responsible, utilize their time, and serve people well, (HIV negative woman)

Another large barrier to treatment uptake and follow-up is the perceived financial burden of treatment for cervical cancer. Many people felt that they would not be able to afford the treatment so they chose to opt-out of screening completely. This was compounded by a death sentence belief regardless of treatment.

When they are diagnosed with [cervical cancer] they are [worried], as cancer is nowadays viewed as a disease for the wealthy, not the poor. (Woman living with HIV)

#### Applying the framework

Our findings demonstrated that some risk factors for HPV further stigmatize the disease, particularly with “early sexual debut” and “multiple sexual partners.” While there is truth to these being risk factors, this enables cultural and community-based judgment and stigma to be projected on the woman who is infected with HPV.

The study found that a combination of drivers of stigma results in stigma manifestations that later impact an individual’s choice to seek screening or treatment. In the case of ambiguous messaging and lack of knowledge, both lead to misinformation, especially within educational interventions. This misinformation is correlated with gossip and prejudice towards certain individuals, which reinforces stereotypes and anticipated stigma. These discourage that individual from both screening and treatment initiatives out of fear or stigma from the community.

A lack of knowledge due to insufficient reproductive health education contributed to a fear of death, a manifestation of internalized stigma associated with hopelessness. In the transcripts, many women associated HPV with death because of a lack of knowledge regarding treatment options and their benefits. This eventually leads to a lack of screening and treatment due to both hopelessness and denial in coming to terms with their diagnosis.

The transcripts revealed that other misinformation, especially regarding circumcision, impacts cervical cancer risk behaviors. While circumcision decreases the risk of HPV transmission, we found that many women have linked sexual relationships with circumcised partners to being without any risk of contracting HPV. This misinformation led to both denial and the avoidance of safe sex practices, which in turn furthers the risk of contracting HPV.

## Discussion

Using qualitative data from women and health care providers in a high HIV prevalence area of western Kenya, we developed a framework to describe the interaction between intrinsic risk factors for HPV, societal drivers, and potential manifestations of HPV-related stigma and their potential impact on HPV risk and prevention behaviors. Themes arose throughout the data to suggest potential drivers for HPV-related stigma within this community. These potential drivers could be detrimental to the vaccine initiatives mentioned earlier in this paper. Key drivers included lack of awareness and misconceptions about HPV and cervical cancer, stigma associated with HPV risk behaviors, a general fear of death, and potential stigmatizing attitudes from health care providers. Many women felt that HPV would “mark” a woman as having HIV, and some women living with HIV felt that a diagnosis of HPV would be overwhelming after learning to live with HIV, suggesting the possibility of intersectional stigma.

The manifestations of stigma resulting from these potential drivers may be associated with an increase in cervical cancer risk behaviors. One of the anticipated manifestations of stigma, mistreatment by providers, would result in an avoidance of screening due to a lack of trust in providers. Fear of death, a manifestation of internalized stigma, is associated with hopelessness, especially for women with both HIV and HPV. This feeling of hopelessness acts as a barrier that, when compounded by the financial burden of treatment, discourages women from accessing medical services. One strategy to address stigma is patient-centered care to ensure that women are comfortable with utilizing services in their area. Patient-centered care would include HPV testing via self-collection and peer-to-peer outreach and education, with a focus on stigma-reducing messaging and increased availability of treatment services. However, while HPV self-testing may be less stigmatizing, women who test positive will require further evaluation with speculum exams and possibly treatment. While this study focused on screening, treatment must also be tailored to patient needs and incorporate stigma responsive strategies, such as including counseling and peer support. The adoption of patient-centered care practices can potentially lead to an increase in HPV and cervical cancer screening and treatment uptake, and follow-up. Improving health outcomes for women with HPV and cervical cancer begins with addressing and mitigating the effects of stigma.

This study highlights the need for further research into the character and effects of reproductive health stigma on health outcomes, especially for women in marginalized communities. Prior epidemiological studies have demonstrated cervical cancer to be the leading cause of female cancer deaths in Kenya, however, few studies have researched the potential intersection of high cervical cancer mortality rates and HPV stigma in LMICs [2]. In this study, it was commonly seen that many women would avoid being screened altogether due to their fear of a potential HPV diagnosis, which they associated with cervical cancer and therefore, death. One comparable study reported that Kenyan men avoided HIV testing and treatment largely due to a fear of a positive HIV test result, which was linked to stigmatization [21]. Similarly, another study in South Africa reported that 33% of participants, both those with previous testing experience and new testers, reported fear of death as a barrier to HIV testing [22]. With HPV and HIV sharing similar manifestations of stigma and health outcomes, the presence of intersectional stigma should be a topic for further research. This will allow stakeholders to better understand how to develop successful and culturally appropriate interventions along with evidence-based educational tools to increase screening rates and treatment uptake.

A strength of this study is that it is the first, to our knowledge, to analyze the impact of stigma specific to HPV care in western Kenya. In addition, the study design was diverse in interviewing participants and stakeholders. This provided a unique perspective on the relationships of HIV, HPV, and cervical cancer stigma in a healthcare setting, as FACES patients with and without HIV gave both patient and community perspectives. Moreover, the multiple types of health care workers included in this study were able to provide further insight on their patients’ lived experiences, thus strengthening the perspectives and testimonies of the study participants living with HIV and/or HPV.

Limitations included a lack of available HPV-positive participants to interview leading to few personal accounts of enacted or experienced HPV and cervical cancer stigma and discrimination. Although we observed common themes emerging among the participants, the small number may limit the generalizability to the larger community. In addition, the findings may be biased, as the women willing to participate may not reflect the overall mentality of the community and may represent women less affected by stigma. Participants most affected by HPV or cervical cancer stigma may have been deterred from the study, therefore creating an underestimation of HPV and cervical cancer stigma within the study population. Although most participants had some knowledge of cervical cancer, HPV knowledge was particularly limited. The extent to which fear and stigma are impacted by lack of knowledge, limited exposure, or education is unclear from this study. As HPV testing, and therefore messaging and awareness becomes more widely available, this must continue to be explored. Finally, as this was an exploratory study, we did not quantitatively measure stigma related to HPV and its possible impact on screening or follow-up.

## Conclusion

The results from the in-depth interviews and existing literature on HPV risk and risk factors for cervical cancer allowed for the development of a framework on the drivers, manifestations, and impacts of HPV and cervical cancer-related stigma. This study suggested that stigma related to HPV, cervical cancer, or both could impact women’s uptake of cervical cancer screening and treatment services. This understanding of the drivers, pathways, and results of HPV and cervical cancer-related stigma will allow us to develop tools to examine the impact of stigma on care utilization leading to the development of educational tools, messaging, and care delivery models to ensure patient-centered care services for cervical cancer prevention.

## Supplementary Information

**Additional file 1**. The full list of interview questions utilized during in-depth interviews with women living with HIV, HIV-negative women, community health volunteers (CHVs), and health care providers in Kisumu, Kenya in 2019.

**Additional file 2**. The codes and definitions used to qualitatively analyze interview transcripts with study participants.

## Data Availability

Per the Kenya Medical Research Institute (KEMRI) guidelines, the datasets and interview transcripts used and/or analyzed during this study are available from the corresponding author upon reasonable request
