# Supplementary material for: Developing a framework to describe stigma related to cervical cancer and HPV in western Kenya
Source: BMC Womens Health. 2022 Feb 11;22:39. doi: 10.1186/s12905-022-01619-y (PMC8832662; doi:10.1186/s12905-022-01619-y)
Supplement: Supplementary file 1 — Additional file 1. The full list of interview questions utilized during in-depth interviews with women living with HIV, HIV-negative women, community health volunteers (CHVs), and health care providers in Kisumu, Kenya in 2019. [file 12905_2022_1619_MOESM1_ESM.pdf]

<u>APPENDIX A</u>

<u>In-depth Interview with Current HIV Negative Women</u>

Thank you for participating in this study to explore what women think about cervical cancer and human papillomavirus. We appreciate your honesty and willingness to assist with this important research. First, could you tell me a little bit about yourself?

HPV and Cervical cancer:

1. As you know, I want to spend time with you today talking about cervical cancer. To begin, please tell me what you know or have heard about cervical cancer

   PROBE: If they have never heard of cervical cancer, explain that it is cancer of the womb, causes bleeding, weight loss. Some may not be familiar with the name, but know the disease. If completely unfamiliar, can exclude from the study.

   a. What do you think causes cervical cancer?
   b. Do you think there is any way to prevent cervical cancer? How?
   c. Do you think you are at risk? Why or why not?

2. I would like to understand what you know about human papillomavirus, or HPV. What have you heard about HPV?

   NOTES: If they have never heard of HPV, explain that it is the virus that causes cervical cancer, is sexually transmitted, but can go completely unnoticed by women unless they screen for cervical cancer.

   a. What is the difference between HPV and cervical cancer?
   b. What factors put someone at risk for cervical cancer? And for HPV?
   c. What do you think/would you think about someone diagnosed with HPV? What if you were diagnosed with HPV?
   d. Does your husband/partner know about HPV? What types of things has he said anything about it?

3. What is the purpose of cervical cancer screening? What different types of screening have you heard of?

   NOTES: May need to explain purpose and types of screening

   a. Have you ever been screened
      i. If no, why not? Prompts: cost? Not offered? Did you understand the counseling? Was the provider respectful? Was a pelvic exam necessary?
      ii. If yes, why? Prompts: perception of risk? Free? Friends told me?
      iii. Did your friends and family help you make that decision? What did they know about the screening?
      iv. Does your husband/partner know about screening? If you spoke with him, what kind of things did he say about it?
   b. Who needs screening? Do you believe that you should be screened?
   c. Have you ever had a pelvic exam? (May need to explain). For what purpose? What were your thoughts around the exam? What do women that you know think about pelvic exams?

4. What kind of people are most at risk to test positive for HPV?

   a. Why do you think that such people are likely to get HPV and eventually cervical cancer?
   b. What can women do to have control over their risk to develop cervical cancer?

5. Do you know anyone who has had cervical cancer?

   For each person:

   a. Tell me about their experiences getting diagnosed.

b. Who have they shared this news with? What was the response?
c. Tell me about their interactions with CHVs and health care professionals.
d. How is this person seen by others? Treated by others?
e. Do you know how their life changed after their diagnosis? How have they coped with their diagnosis?
f. Do you know anyone who has been diagnosed with HPV? Tell be about their experiences.

HIV and HPV:

6. When was the first time you had an HIV test?
7. What led you to get tested for HIV?
8. What was your experience? Did you have any negative experiences? Positive experiences?
9. Did your friends or family know that you were tested? How did you share that information with them?
10. Do you know anyone who tested positive for HIV? What is your interaction like with them?
11. In your own experience, are people living with HIV treated differently than those who are HIV negative? Describe the instances when you have encountered this situation.
12. Do you think people living with HIV are responsible for their HIV diagnosis? Why or why not?
13. How would you feel if you tested positive for HIV?
14. You just told me how you would feel about getting a positive HIV test. Now consider that you got a positive test for HPV. How would you feel about that?
15. Do you feel that there are differences between testing positive for HPV and positive for HIV?
    a. How is it different or the same to have HPV compared to HIV?
    b. If you haven't already screened for cervical cancer, would you want to do that by getting tested for HPV?
16. How would diagnosis of HPV influence your professional life? Your social life? Your family life/relationship? And your romantic life (i.e. sexual relationship)?

Decision making process in health care:

17. Who in your life influences your decision to go to the doctor, clinic or hospital?
    a. Prompt: Spouse, religious leaders, family, friends, and neighbors?
    b. What kind of influence have they had?
    c. What would your partner say about you getting screened (or another person or another screening method) (time commitment as a wife or a mother)
18. Please describe your experiences in healthcare settings in general. What about regarding women's health services, such as family planning or maternity services?
    a. Do you feel like you are respected?
    b. Have you ever been made to feel shame, worthlessness or embarrassment at a health care visit? Please describe these situations.
19. What would make it easier for you and your friends to go for women's health services? (What makes it difficult?)

Challenges associated with illnesses:

20. What challenges do you think people with HIV have in their daily lives?
21. Do you feel that HIV care and support services are sufficient for those who are HIV positive? Why or why not?
22. What challenges do you think people who have screened positive for HPV have in their daily lives?
23. What challenges do you think people with cervical cancer have in their daily lives?

<u>In-depth Interview with Women Living with HIV</u>

Thank you for participating in this study to explore what women think about cervical cancer and human papillomavirus. We appreciate your honesty and willingness to assist with this important research. First, could you tell me a little bit about yourself?

HPV and Cervical cancer:

1. As you know, I want to spend time with you today talking about cervical cancer. To begin, please tell me what you know or have heard about cervical cancer

   PROBE: If they have never heard of cervical cancer, explain that it is cancer of the womb, causes bleeding, weight loss. Some may not be familiar with the name, but know the disease. If completely unfamiliar, can exclude from the study.

    a. What do you think causes cervical cancer?
    b. Do you think there is any way to prevent cervical cancer? How?
    c. Do you think you are at risk? Why or why not?
2. I would like to understand what you know about human papillomavirus, or HPV. What have you heard about HPV?

   NOTES: If they have never heard of HPV, explain that it is the virus that causes cervical cancer, is sexually transmitted, but can go completely unnoticed by women unless they screen for cervical cancer.

    a. What is the difference between HPV and cervical cancer?
    b. What factors put someone at risk for cervical cancer? And for HPV?
    c. What do you think/would you think about someone diagnosed with HPV? What if you were diagnosed with HPV?
    d. Does your husband/partner know about HPV? What types of things has he said anything about it?
3. What is the purpose of cervical cancer screening? What different types of screening have you heard of?

   NOTES: May need to explain purpose and types of screening

    a. Have you ever been screened
        i. If no, why not? Prompts: cost? Not offered? Did you understand the counseling? Was the provider respectful? Was a pelvic exam necessary?
        ii. If yes, why? Prompts: perception of risk? Free? Friends told me?
        iii. Did your friends and family help you make that decision? What did they know about the screening?
        iv. Does your husband/partner know about screening? If you spoke with him, what kind of things did he say about it?
    b. Who needs screening? Do you believe that you should be screened?
    c. Have you ever had a pelvic exam? (May need to explain). For what purpose? What were your thoughts around the exam? What do women that you know think about pelvic exams?
4. What kind of people are most at risk to test positive for HPV?
    a. Why do you think that such people are likely to get HPV and eventually cervical cancer?
    b. What can women do to have control over their risk to develop cervical cancer?
5. Do you know anyone who has had cervical cancer?

   For each person:

    a. Tell me about their experiences getting diagnosed.

b. Who have they shared this news with? What was the response?
c. Tell me about their interactions with CHVs and health care professionals.
d. How is this person seen by others? Treated by others?
e. Do you know how their life changed after their diagnosis? How have they coped with their diagnosis?
f. Do you know anyone who has been diagnosed with HPV? Tell me about their experiences.

HIV and HPV:

1. When were you diagnosed with HIV?
2. What led you to get tested in the first place?
3. What was your experience? How did you react to the diagnosis? Did you have any negative experiences? Positive experiences? Did you disclose your positive HIV status to anyone at the time of diagnosis?
4. Did you disclose to anyone about your diagnosis? (Family, friends, partner, etc.). How did you share that information with them?
    a. Was it difficult to disclose this information with them?
        i. Were you afraid or what they might say after hearing the news?
        ii. Did you feel responsible for your HIV diagnosis?
5. What did you know about HIV before diagnosis?
6. What do you wish you had know about HIV before diagnosis?
7. In your own experience, are people living with HIV treated differently than those who are HIV negative? Describe the instances when you have encountered this situation.
8. Do you feel that you're treated differently because of your HIV status? Describe the instances when you felt this way.
9. Do you think people living with HIV are responsible for their HIV diagnosis? Why or why not?
10. Do you feel that there are differences between testing positive for HPV and positive for HIV?
    a. How is it different or the same to have HPV compared to HIV?
    b. How would it feel to *know* that you don't have HPV compared to having HIV?
    c. If you haven't already screened for cervical cancer, would you want to get tested for HPV?
11. What is the best way to teach those with HIV about HPV and cervical cancer? (Modes of teaching/education, content, etc.)
12. How has your positive HIV status impacted your life in the social and professional areas?
13. How would diagnosis of HPV or cervical cancer influence your professional life? Your social life? Your family life/relationship?
    a. As someone who already has HIV, how would it feel learning that you had HPV?
    b. As someone who already has HIV, how would it feel learning that you had cervical cancer?
    c. Do you feel that you will have adequate support and counseling to receive care in HIV and HPV or cervical cancer?

Decision making process in health care:

14. Who in your life influences your decision to go to the doctor, clinic or hospital?
    a. Prompt: Spouse, religious leaders, family, friends, and neighbors?
    b. What kind of influence have they had?
    c. What would your partner say about you getting screened (or another person or another screening method) (time commitment as a wife or a mother)

15. Please describe your experiences in healthcare settings in general. What about regarding women's health services, such as family planning or maternity services?
    a. Do you feel like you are respected?
    b. Have you ever been made to feel shame, worthlessness or embarrassment at a health care visit?
16. What would make it easier for you and your friends to go for women's health services?
17. What makes it difficult for you and your friends to go for women's health services?

Challenges associated with illnesses:

18. What challenges do you face in your daily life?
19. Do you feel that the HIV care and support services are sufficient? Why or why not?
20. What challenges do you think other people with HIV have in their daily lives?
21. What challenges do you think people with HPV have in their daily lives?
22. What challenges do you think people with cervical cancer have in their daily lives?

<u>In-depth Interview with HIV care providers</u>

Thank you for participating in this study to explore what women think about cervical cancer and human papillomavirus. We appreciate your honesty and willingness to assist with this important research. First, could you tell me a little bit about yourself?

HIV care:

1. What made you choose to work in HIV care?
2. Tell me about your job. What HIV/AIDS related services do you provide? (Clinical care, counseling, testing, dispensing drugs, support group services, etc.)
3. What do you like about your job? What are some challenges that you face in your job?
4. How long have you worked in HIV/AIDS care?
5. Did you have formal HIV training?
    a. What topics were covered? (HIV prevention, HIV counseling, HIV stigma, antiretroviral therapy, adherence, prevention of mother-to-child transmission, nutrition, etc.)
    b. Have you ever had training in counseling? In reproductive or women's health care? What did you like or dislike about these topics?
    c. Have you been trained to do pelvic exams? How do you feel about being trained to do this? How do you feel about carrying out these exams in the clinic?
6. Let's talk about how clients respond to testing and receiving their diagnosis.
    a. NOTE: If s/he reports that testing is part of their job: what is the most frequent or typical way that you offer HIV testing? Are there typical circumstances in which this occurs? Do you follow guidelines to do so?
    b. How do women respond to an offer of an HIV test?
    c. What are some reactions when people receive their HIV diagnosis? What are some fears or concerns you have seen addressed? Can you think of examples of helping people work through their fears?
    d. Do you think women have different reactions to an HIV diagnosis compared to men? Do you think people in your community react differently to women getting an HIV-diagnosis, compared to men?

7. Have you ever seen or heard of people who have been badly treated because they have HIV? Describe what happened.

I would like to talk to you about what you have observed for patients with HIV are treated in a clinical setting.

8. Have you seen any instances in which HIV providers seemed uncomfortable with a patient because of his or her HIV status? Describe what happened. What do you think caused the discomfort?
    a. How concerned are you about getting infected with HIV in the course of your work?
9. Have you seen HIV providers' gossip about someone's HIV status? Describe what happened.
10. Have you seen HIV positive patients being ignored or receive less care than other patients? Describe what happened. Do you
11. Have you seen providers deny care to HIV positive patients? Describe what happened.
12. Have you seen providers treat people with HIV differently than people who do not have HIV? Describe what happened.
13. Have you seen any HIV positive patients being treated with disrespect or abused? Describe. Is this different between men and women?
14. Do you think that people living with HIV are responsible for their HIV status?

Working with women living with HIV:

15. Describe your comfort providing care for HIV positive women. What are some of the health concerns that are different for women compared to men? What other factors impact their engagement with care? Adherence? Health outcomes?
16. Do you think others disrespect you for your proximity to HIV positive women?
17. Are you aware of any instances of women feeling ashamed in receiving care for their HIV status?
18. Do you think women with HIV feel supported both medically and emotionally with the HIV care services provided to them?
19. Do you think people living with HIV should disclose their HIV status? Who do you think they should tell? What challenges do they face in disclosing their status?
20. Have HIV positive women told you that they experienced any of the following as a result of their HIV status? (Abandoned by a spouse/partner, abandoned by other family members, made fun of by others, physically assaulted, fired from their job)

Cervical cancer and screening services:

21. Could you tell me what you know about cervical cancer?
22. Could you tell me what you know about HPV? How is it transmitted to people? Do you have any experience in providing HPV testing?
23. Do you provide cervical cancer screening to your patients? What kind?
    a. Do you think this is an important service? Why?
    b. Do patients typically ask for the screening, or is it offered to them?
    c. When during a client's care do you offer cervical cancer screening?
24. What have you learned about the kinds of people most likely to get HPV or cervical cancer?
    a. Why do you think that such people are likely to get HPV or cervical cancer?
25. Do you provide cervical cancer screenings to clients in your clinic? What kind? Is there a different criteria for those who HIV-positive vs. HIV-negative?
    a. Do you think this is an important service? Why?
    b. Do patients typically ask for the screening, or is it offered to them?
26. What are different considerations you would have for a woman who tested HPV positive compared to HPV negative?
27. How do patients with cervical cancer typically cope with the diagnosis?

28. Do the patients who are diagnosed with cervical cancer feel that they have adequate support from their family? Friends? Clinic staff?

HIV and HPV:

29. What are some of the reasons that people wait or decide not to seek out testing for HIV?
    a. What about treatment for HIV?
    b. What types of things would make a health care provider more trustworthy?
30. What are some of the reasons that people wait or decide not to seek out testing for HPV?
    a. What about treatment for HPV?
31. Do you see any similarities in the interactions with which care providers treat women with HIV diagnosis rather than HPV diagnosis? What about a diagnosis of cervical cancer?
32. Do you have colleagues who refuse to treat or provide less care to women infected with HPV? What reasons are there?

Challenges associated with illnesses:

33. What challenges do you think people with HIV in their daily lives?
34. Do you feel that HIV care and support services are sufficient for those who are HIV positive? Why or why not?
35. What challenges do you think people with HPV in their daily lives?
36. What challenges do you think people with cervical cancer in their daily lives?
37. What do you are the major barriers that people with HIV have in access to care? What about people with HPV? And people who have cervical cancer?
38. What do you think are the major barriers that people with HIV have in getting treatment?
    a. What about people with HPV?
    b. And people who have cervical cancer?
    c. Can you describe the differences in treatment options. What might cause them to not get treated?
39. What do you think hinders people in this community from being tested for HIV?
40. What could be done to improve access to testing for those who want it?
41. What are the main challenges that staff face in providing HIV care/counseling?

<u>**In-depth Interview with Community Health Volunteers**</u>

Thank you for participating in this study to explore what women think about cervical cancer and human papillomavirus. We appreciate your honesty and willingness to assist with this important research. First, could you tell me a little bit about yourself?

The role of CHV and CHV training:

1. What health services/counseling do you provide in your role as a community health volunteer? What services do you enjoy providing? Why? Which do you enjoy less and why?
2. How long have you worked as a community health volunteer?
3. Have you had formal HIV training? Women's health care training? What kinds?
4. Have you had any education or training in HPV and/or cervical cancer? If so, what was covered during the training? How long was the training?

Working with women living with HIV:

5. Do you feel comfortable providing care for HIV positive women?

6. How concerned are you about getting infected with HIV in the course of your work?
7. Do you think others disrespect you for your proximity to HIV positive women?
8. Are you aware of any instances of women feeling ashamed in receiving care for their HIV status? What are some instances you have seen?
9. Do you think that people living with HIV are responsible for their HIV status?
10. Do you think women feel supported both medically and emotionally with the HIV care services provided to them? Where does the responsibility rely on? Do you think HIV Pos women are more responsible for the infection
11. Do you think people living with HIV should disclose their HIV status? Who do you think they should tell apart from their partners? What challenges do they face in disclosing their status?
12. Have HIV positive women told you that they experienced any of the following as a result of their HIV status? (Abandoned by a spouse/partner, abandoned by other family members, made fun of by others, physically assaulted, fired from their job)
13. Have you seen any HIV positive patients being treated with disrespect or abused? Describe what happened.
14. Have you seen HIV positive patients being ignored or receive less care than other patients? Describe what happened.
15. Have you seen providers deny care to HIV positive patients? Describe what happened.

Comfort with the provision of HPV and cervical cancer care:

16. Could you tell me what you know about cervical cancer?
17. What do you know about HPV?
18. How comfortable are you in discussing HPV or cervical cancer with patients? What are things that make it more or less comfortable?
    a. Do you feel comfortable with your knowledge of HPV and/or cervical cancer?
    b. Do you feel that women are embarrassed to talk about HPV?
19. Do you feel comfortable providing follow-up care/referral for women who screen positive for HPV?

Experiences with HPV and cervical cancer care:

20. Have you had any patients who tested positive for HPV? If so, could you share your experience(s)?
21. Have you had any patients who was diagnosed with cervical cancer? If so, could you share your experience(s)?
22. Do you think people find it easier to talk to you about HPV and cervical cancer with you than with health care providers in clinics?
23. What are some of the reasons that people wait or decide not talk to you (or another provider) about their diagnosis if they are diagnosed with cervical cancer ?
24. If you have talked to patients about HPV or cervical cancer, what are some of the ways you talk about HPV or cervical cancer? Do you have a "script" to counsel women about screening?
    a. How about for treating cervical cancer?
25. If you have not talked to patients about HPV or cervical cancer, how would start a conversation about HPV and cervical cancer? What would you discuss with them? (Mode of transmission, rescreening timeline, treatment, etc.)
26. What are some of the counseling support that currently exist? Do you think it's sufficient?
27. Tell me about concerns women communicate with you regarding screening.
    a. What about women who test positive for HPV?

28. What is the general public response to those diagnosed with cervical cancer? What about HPV?
29. Have you seen any HPV positive and/or cervical cancer patients being treated with disrespect or abused? Describe what happened.
30. Have you seen HPV positive and/or cervical cancer patients being ignored or receive less care than other patients? Describe what happened.
31. Have you seen healthcare providers deny care to HPV positive and/or cervical cancer patients? Describe what happened.
32. Do you think women living with HPV are responsible for their HPV status?
33. Do you think women who have cervical cancer are responsible for their diagnosis?
34. Are there reasons people may feel opposed to supporting those with cervical cancer?
35. What kind of people are at risk for HPV or cervical cancer? Why do you think that such people are likely to get cervical cancer?
36. Are there reasons you may feel opposed to supporting/treating those infected with HPV?
37. How would you describe the extent of religious beliefs in the community you serve?
    a. Are there certain beliefs which guide your own practices?
    b. In what ways would they influence your care for women with HIV? What about HPV? Cervical cancer?

Challenges associated with illnesses:

38. What challenges do you think people with HIV have in their daily lives?
39. Do you feel that HIV care and support services are sufficient for those who are HIV positive? Why or why not?
40. What challenges do you think people with HPV have in their daily lives?
41. What challenges do you think people who are diagnosed with cervical cancer have in their daily lives?
42. What do you are the major barriers that people with HIV have in access to care? What about people with HPV? And people who have cervical cancer?
43. What do you think are the major barriers that people with HIV have in getting treatment?
    a. What about people with HPV?
    b. And people who have cervical cancer?
    c. Can you describe the differences in treatment options? What might cause them to not get treated?
