# Supplementary material for: Developing a framework to describe stigma related to cervical cancer and HPV in western Kenya
Source: BMC Womens Health. 2022 Feb 11;22:39. doi: 10.1186/s12905-022-01619-y (PMC8832662; doi:10.1186/s12905-022-01619-y)
Supplement: Supplementary file 2 — Additional file 2. The codes and definitions used to qualitatively analyze interview transcripts with study participants. [file 12905_2022_1619_MOESM2_ESM.pdf]

APPENDIX B

Name	Description
**Cultural Traditions and Norms**	Practices or beliefs held by the community related to culture or religion that may influence screening, treatment, or education surrounding HIV, HPV, or cervical cancer.
Alternative and Traditional Medicine	Use of medicine or practices outside of the modern health care system.
Myths surrounding HIV Positive Status	Cultural stigma about these diseases based on myths about disease (i.e. witchcraft causing HIV).
Young Girls Frowned Upon for Seeking Family Planning	Cultural stigma against family planning.
**External Support**	Presence or lack thereof of physical, emotional, or financial support given to an individual with HIV, HPV, or cervical cancer.
Community	Physical, emotional, or financial support from community members (not including immediate or extended family) of the HIV, HPV, or cervical cancer positive individual.
Other Family Members	Physical, emotional, or financial support from immediate or extended family (not including spouse or domestic partner) of the HIV, HPV, or cervical cancer positive individual.
Partner	Physical, emotional, or financial support from a domestic partner or spouse of the HIV, HPV, or cervical cancer positive individual.
**General Misconceptions - HIV**	Misconception or misinformation regarding aspects of HIV including screening, treatment, transmission, cause, etc.
Misconceptions about screening and treatment	Misbelief about the screening or treatment for HIV based on faulty understanding or miscommunication.
Misinformation about disease	Incorrect knowledge about the disease, methods of transmission, causes of disease, etc.
**General Misconceptions - HPV and CC**	Misconception or misinformation regarding aspects of HPV or cervical cancer including screening, treatment, transmission, cause, etc.

Misconceptions about screening and treatment	Misbelief about the screening or treatment for HPV or cervical cancer based on faulty understanding or miscommunication.
Misinformation about disease	Incorrect knowledge about the disease, methods of transmission, cause of disease, or believing that HPV and cervical cancer are the same.
**Health Services**	Experiences of individuals with HIV, HPV, or cervical cancer in healthcare settings.
Attitudes towards providers	Patient's feelings towards providers (i.e. mistrust, trust, comfort, discomfort, gratitude, etc.).
Behaviour of Providers	Provider's attitudes, behaviors, or actions, typically interpreted negatively, towards a patient.
Experience with counseling	Negative or positive experiences with counseling or whether they received counseling for their disease at all.
Perceived Quality of Health Services	Observations about the quality of the health services: treatment during appointments, time spent waiting, clean instruments, etc.
**HIV Coping Strategy or Process**	The methods that an individual with HIV uses to cope or come to terms with their HIV status.
Acceptance	Coming to terms with HIV status, process of acceptance.
Child Tested Negative	Their child testing negative may inspire the individual to move on and live a healthy life so that they may manage their disease; a reason to live.
Faith Based or God Reference	Individuals turning to religion or God to cope with HIV status.
Other PLWH	Relieved that the other individuals appears healthy and/or they can't tell the individual is HIV positive.
**HIV Disclosure**	Experiences from an HIV individual disclosing their status
Disclosure in Discordant Partners	Events that may transpire when disclosing status amongst discordant couples (abandonment by the male partner or HIV negative partner).
Negative Outcome	Facing abandonment by a partner, shunned by the community, lack of support, segregation, or loss of job.

Positive Outcome	Could be met with support from family members and community, help with taking medications and doctor visits.
**HIV Fear**	Types of fear associated with receiving an HIV positive diagnosis.
Death	Anticipated fear of death from either HIV or AIDS due to a HIV positive test result.
Financial Strain	Anticipated or experienced fear of financial burden or restraint due to a HIV positive test result.
Outcomes of Disclosure	Anticipated or experienced fear of the consequences associated with revealing a HIV positive test result to others.
Positive Diagnosis	Anticipated or experienced fear of receiving a HIV positive test result
**HIV Internalized Negative Attitudes**	General negative feelings surrounding an individual's HIV positive status or diagnosis.
Bitterness or Anger towards others	Anger towards oneself or others (including the individual responsible for transmitting HIV) for a HIV positive result.
Denial	Refusal to believe a positive HIV test result.
Depression	General feelings of despair, hopelessness, anti-social, or unhappiness.
Self-Blame	Shame of a HIV positive result. Guilt, blame, or regret of the actions or individuals responsible for contracting HIV.
Worrying over others' opinions	Embarrassment or worry over others' opinions or beliefs of the HIV positive individual.
**HIV Internalized Positive Attitudes**	General positive feelings surrounding an individual's HIV status or diagnosis.
Feeling gratitude or relief	Feeling relieved for receiving a negative HIV test result.
Wanting to be a role model	Despite a positive or negative HIV test result, the individual is fueled to be a role model in their community and help other people.
**HIV Screening**	Factors that influence an individual's decision to be screened for HIV.
Barriers to screening or treatment	Physical, emotional, or financial hardships while attempting to be screened or treated for HIV (either experienced by the individual or a member of the community).

Drivers to screening	Lack of hardships or encouragement (either physical, emotional, or financial) while attempting to be screened for HIV. Either experienced by the individual or a member of the community.
Who should get screened	Mentions of categories or characteristics of the “type” of individual that may be or is encouraged to be screened for HIV.
**HPV and Cervical Cancer Coping Strategy or Process**	The methods that an individual with HPV or cervical cancer uses to cope or come to terms with their condition.
Acceptance	Coming to terms with HPV or cervical cancer status, process of acceptance.
Faith Based or God Reference	An individual turning to religion or God to cope with their HPV or cervical cancer status.
**HPV and Cervical Cancer Fear**	Types of fear associated with receiving an HPV or cervical cancer diagnosis.
Death from Cervical Cancer	Anticipated fear of death from cervical cancer due to a HPV or cervical cancer positive test result.
Embarrassment or Discomfort with Male Providers	Anticipated or experiences fear of interacting with male health professionals or community health workers. Also includes feelings of embarrassment towards the screening procedure.
Financial Strain	Anticipated or experienced fear of financial burden or restraint due to a HPV or cervical cancer positive test result.
Outcomes of Disclosure	Anticipated or experienced fear of the consequences associated with revealing a HPV or cervical cancer positive test result to others.
Pain of Screening Procedure	Anticipated or experiences fear of pain or discomfort from the HPV or cervical cancer screening procedure.
Positive Diagnosis	Anticipated or experienced fear of receiving a HPV or cervical cancer positive test result.
**HPV and Cervical Cancer Internalized Negative Attitudes**	General negative feelings surrounding an individual’s HPV or cervical cancer diagnosis.
Bitterness or Anger towards others	Anger towards oneself or others (including the individual responsible for transmitting HPV) for an HPV positive result.
Denial	Refusal to believe a positive HPV or cervical cancer test result.

Depression	Feelings of despair, hopelessness, anti-social, unhappiness, etc
Self-Blame	Blaming self for contracting HPV or getting cervical cancer
Worrying over others' opinions	Embarrassment or worry over others' opinions or beliefs of the HPV positive individual.
**HPV and Cervical Cancer Screening**	Factors that influence an individual's decision to be screened for HPV or cervical cancer.
Barriers to screening or treatment	Physical, emotional, or financial hardships while attempting to be screened or treated for HPV (either experienced by the individual or a member of the community).
Drivers to screening	Lack of hardships or encouragement (either physical, emotional, or financial) while attempting to be screened for HIV (either experienced by the individual or a member of the community).
Experiences with screening	Negative interactions with healthcare workers during HPV or cervical cancer screenings including counselling, procedure, treatment, etc experienced either by the individual or the community.
Who should get screened	Mentions of categories or characteristics of the "type" of individual that may be or is encouraged to be screened for HPV.
**HPV Internalized Positive Attitudes**	General positive feelings surrounding an individual's HPV or cervical cancer diagnosis.
Feeling gratitude or relief	Feeling relieved for receiving a negative HPV test result.
Wanting to be a role model	Despite a positive or negative HIV test result, this fuels them to be role models in their communities and help other people.
**Impact of Education on Women**	Factors related to education that influence a woman's knowledge or decisions, including screening and treatment, regarding HIV, HPV, or cervical cancer.
Clarification of Myths	To clear up incorrect assumptions or myths about people with HIV, HPV, or cervical cancer held by the community.
Motivating Factor for Screening, Receiving or Continuing with Treatment	Pushes women to get screened and/or to receive or continue with treatment.
Sense of Empowerment	Empowerment based on knowledge (not necessarily action-taking). Empowering women to take control of their own health.

**Memorable Quotes**	Any memorable quotes that may resonate with us and can be used for the paper.
**Men**	The influence and role that men play in a women's decision to pursue healthcare services for HIV, HPV, or cervical cancer.
Blame	Female believes male partner is at fault for cause of infection or male partner believes female partner is at fault for cause of infection.
Discordant Status (HIV)	Actions associated with being a discordant couple (i.e. abandonment)
Gender-Based Violence	Experience of IPV, domestic violence, and/or gender-based violence (typically where the male partner is the aggressor)
Lack of Support	Passively supporting, disagreeing or telling the individual not to get screened, abandonment.
**Stigmatizing Attitudes towards HPV and Cervical Cancer**	Prejudices surrounding an individual's HPV or cervical cancer status that can lead to differing attitudes, beliefs, or actions towards the individual.
How they or others discriminate against people with HPV or Cervical Cancer	Experienced or anticipated stigma either by the individual or the community towards individuals with HPV or cervical cancer (involves an action).
Perceptions of people with HPV or Cervical Cancer (faithfulness and promiscuity)	Negative perceived characteristics, assumptions, or prejudices towards individuals with HPV or cervical cancer either by the individual or the community such as faithfulness or promiscuity.
Stigmatizing Messaging for HPV or Cervical Cancer	Educational messaging that contains misinformation or stigmatizes people with HPV or cervical cancer.
**Stigmatizing Attitudes Towards PLWH**	Prejudices surrounding an individual's HIV status that can lead to differing attitudes, beliefs, or actions towards the individual.
How they or others discriminate against PLWH	Experienced or anticipated stigma either by the individual or the community towards individuals with HIV (involves an action).
Perceptions of PLWH (faithfulness and promiscuity)	Negative perceived characteristics, assumptions, or prejudices towards individuals with HIV either by the individual or the community such as faithfulness or promiscuity.
Stigmatizing Messaging for PLWH	Educational messaging that contains misinformation or stigmatizes people with HIV.
